# Transcriptome analysis reveals the high temperature induced damage is a significant factor affecting the osmotic function of gill tissue in Siberian sturgeon *(Acipenser baerii)*

**DOI:** 10.1186/s12864-022-08969-9

**Published:** 2023-01-03

**Authors:** Shiyong Yang, Datian Li, Langkun Feng, Chaoyang Zhang, Dandan Xi, Hongli Liu, Chaozhan Yan, Zihan Xu, Yujie Zhang, Yunkun Li, Taiming Yan, Zhi He, Jiayun Wu, Quan Gong, Jun Du, Xiaoli Huang, Xiaogang Du

**Affiliations:** 1 Department of Aquaculture, College of Animal Science & Technology, Sichuan Agricultural University, Chengdu, 611130 Sichuan China; 2 College of Landscape Architecture, Sichuan Agricultural University, Chengdu, 611130 Sichuan China; 3 College of Life Science, Sichuan Agricultural University, Ya’an, 625014 Sichuan China; 4 Fisheries Institute, Sichuan Academy of Agricultural Sciences, Chengdu, 610066 Sichuan China

**Keywords:** RNA-seq, Heat stress, Osmoregulation, Gill tissue

## Abstract

**Background:**

Maintaining osmotic equilibrium plays an important role in the survival of cold-water fishes. Heat stress has been proven to reduce the activity of Na^+^/K^+^-ATPase in the gill tissue, leading to destruction of the osmotic equilibrium. However, the mechanism of megatemperature affecting gill osmoregulation has not been fully elucidated.

**Results:**

In this study, Siberian sturgeon (*Acipenser baerii*) was used to analyze histopathological change, plasma ion level, and transcriptome of gill tissue subjected to 20℃, 24℃and 28℃. The results showed that ROS level and damage were increased in gill tissue with the increasing of heat stress temperature. Plasma Cl^−^ level at 28℃ was distinctly lower than that at 20℃ and 24℃, while no significant difference was found in Na^+^ and K^+^ ion levels among different groups. Transcriptome analysis displayed that osmoregulation-, DNA-repair- and apoptosis-related terms or pathways were enriched in GO and KEGG analysis. Moreover, 194 osmoregulation-related genes were identified. Amongst, the expression of genes limiting ion outflow, *occluding* (*OCLN*), and ion absorption, s*olute carrier family 4, member 2 (AE2)* s*olute carrier family 9, member 3 (NHE3) chloride channel 2 (CLC-2)* were increased, while Na^+^/K^+^-ATPase alpha *(NKA-a)* expression was decreased after heat stress.

**Conclusions:**

This study reveals for the first time that the effect of heat stress on damage and osmotic regulation in gill tissue of cold-water fishes. Heat stress increases the permeability of fish’s gill tissue, and induces the gill tissue to keep ion balance through active ion absorption and passive ion outflow. Our study will contribute to research of global-warming-caused effects on cold-water fishes.

**Supplementary Information:**

The online version contains supplementary material available at 10.1186/s12864-022-08969-9.

## Background

Since the 1850s, global warming has aggravated climate change [1], which causes the rise of global average temperature and probability of extreme high-temperature events during summertime [2–4]. In China, extreme high temperature occurs frequently in recent summers, resulting in continuous heat stress in breeding of cold-water fishes. Many studies have documented the significant effects of heat stress on cold-water fishes’ immunity, growth metabolism and behavior [4–7].

Freshwater makes fish hypoosmotic, which allows ions in the blood to passively diffuse into the surrounding freshwater. Internal osmotic pressure equilibrium is the foundation to maintain cells and physiological processes. To maintain the osmotic pressure, fish requires coordination of multiple organs, including gill, intestine, and kidney tissues [8–10]. The gill tissue is the critical organ for osmotic regulation system, which regulates ion exchange with the external environment via ion channel activity and tight connection to maintain osmotic equilibrium [11–18]. Importantly, gill tissue directly contacts with the external environment and, thereby, has more probability to subject to environmental stresses [19]. Previously, the effects of heat stress on morphological changes, damage, and oxidative stress were investigated in gill tissue [20–25]. Recently, studies have shown that heat stress could reduce the osmoregulation core enzyme (Na^+^/K^+^-ATPase) activity in gill tissue [25, 26]. However, how heat stress effects fish’s osmoregulation function is still unclear.

Sturgeon, widely distributed throughout the Northern Hemisphere [27], is an important economic cold-water fish for producing caviar [28]. In 2021, farming sturgeon reached to 104,280 ton in China, accounting for nearly 80% production over the world [29]. The proportion of Siberian sturgeon reached to 34% among the cultured sturgeon species in China. The southwest area of China is quit suitable for the breeding of Siberian sturgeon because of the relative low water temperature through whole year [30]. However, the resent crazy high-temperature weather swept the southwest China, which is taking huge challenge to sturgeon breeding.

Previous studies have showed that the optimal water temperature was around 20 °C for survival and growth of Siberian sturgeon [31, 32]. However, in the past 50 years, the average summer temperature in southwest China is around 24℃, and the highest temperature reaches to or overs 29 °C [33]. In the previous study of our group, the gill tissue of Siberian sturgeon was damaged via heat stress (at 24℃ and 28℃) [20]. However, the damaged mechanism is poorly understood. This study aims to investigate the effect mechanism of heat stress with focus on osmotic equilibrium in Siberian sturgeon, which will contribute to generate strategy against the emerging of heat stress issue in aquaculture.

## Results

### Heat stress induces damage of gill tissue

**Fig. 1:**
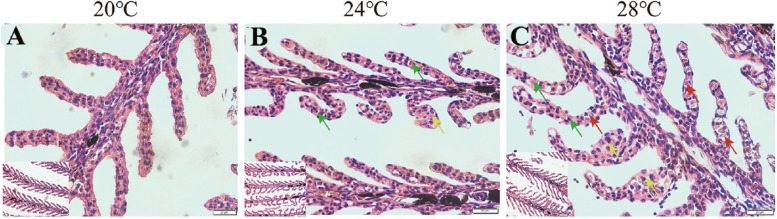
Histopathological changes of gill tissue in Siberian sturgeon. The green, yellow and red arrows indicate denaturation, hyperplasia and necrosis of gill filaments, respectively

The histopathological change of the gill tissue subjected to heat stress was observed via H&E staining (Fig. 1). The results showed that the structure of gill filaments was complete and clearly visible at 20℃, while the epithelial cells of gill filaments occurred degeneration and hyperostosis after heat stress at 24℃ and 28℃, particularly at 28℃ where necrosis was appeared (Fig. 1A).

### Heat stress elevates ROS level of gill tissue

**Fig. 2:**
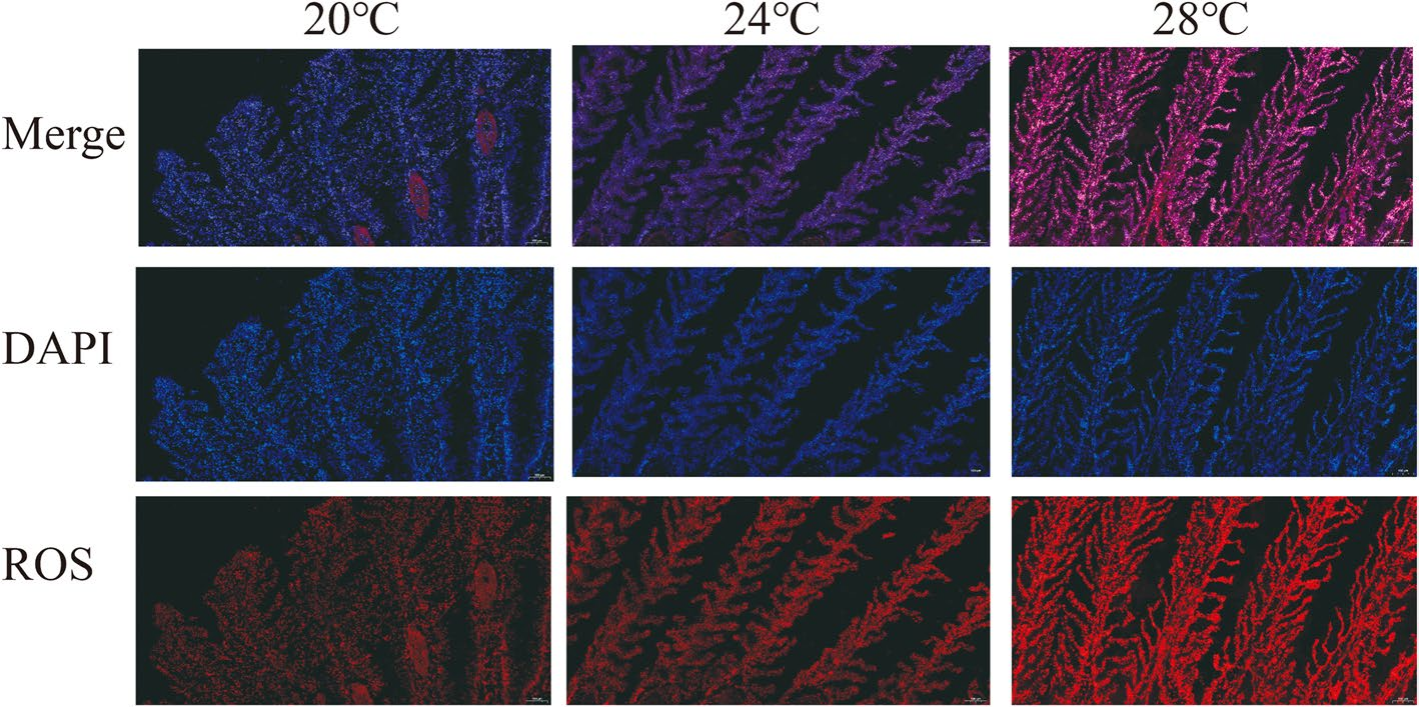
Microscopic images of ROS immunofluorescence in gill filaments of Siberian sturgeon

ROS level of gill tissue was measured via immunofluorescence after heat stress. As shown in Fig. 2, heat stress both at 24℃ and 28℃ induced distinct ROS accumulation in gill filaments. Amongst, the ROS level at 28℃ was significantly high than that at 24℃.

### Heat stress effects plasma Na^+^, K^+^ and Cl^−^ ion contents

**Fig. 3:**
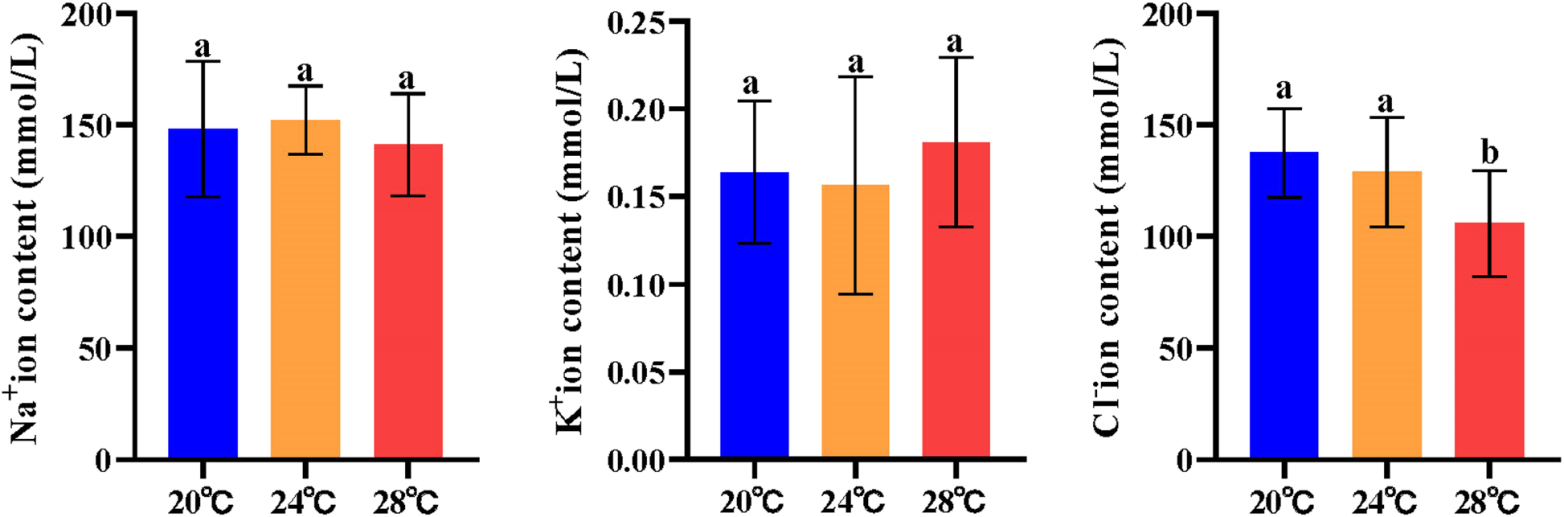
Plasma Na^+^, K^+^ and Cl^−^ ion contents in Siberian sturgeon. Plasma Na^+^ (**A**), K^+^ (**B**) and Cl.^−^ (**C**) ion contents were measured after temperature administration at 20℃, 24℃ and 28℃. One-way ANOVA plus Bonferroni post-tests; different letters indicate statistically significant differences (*p* < 0.05)

Plasma Na^+^, K^+^ and Cl^−^ ion contents were measured after heat stress in Siberian sturgeon (Fig. 3). The results displayed that plasma Na^+^ and K^+^ ion contents was not significantly changed after heat stress at 24℃ and 28℃ than at 20℃. By contrast, plasma Cl^−^ ion content was obviously decreased after heat stress at 28℃ (*p* < 0.05) but no significant change at 24℃ than the control group.

### Library construct, *de novo* assembly and annotation

Nine RNA-seq libraries were constructed from the gill tissue after different temperatures exposures in Siberian sturgeon. All datasets from the Illumina sequencing platform can be found in the National Center for Biotechnology Information (NCBI) Short Read Archive (SRA) database under accession number (PRJNA765171). After sequencing and filtering with raw data, 30 to 44 million clean reads were acquired in each library, whose Q30 values ranged from 94.29% to 96.04% in the libraries. Clean reads from all groups were mapping with the corresponding unigene, the mapping rate is 69.09%-74.76% (Table S1). Total of 172,158 unigenes were acquired from *de novo* assembly. All the unigenes were annotated by five databases, including NR, GO, eggNOG, KEGG and Swiss (Table 1).

**Table 1:** The statistics of annotation

Sample ID	Total protein	NR	GO	eggNOG	KEGG	Swiss
Unigene	172,158	41,905	40,362	31,875	24,456	33,600

### Identification of differentially expressed genes (DEGs), GO and KEGG enrichment analysis

Total 8,570 DEGs (FDR < 0.005, log2 (foldchange) ≥ 2)were identified in 24℃ vs 20℃group, containing 4,593 up-regulated genes, and 4,275 down-regulated genes (Fig. S2 A-B). In 28℃ vs 20℃ group, 11,835 DEGs were identified, containing 5,950 up-regulated genes and 5,885down-regulated genes (Fig. S2 C-D).

**Fig. 4:**
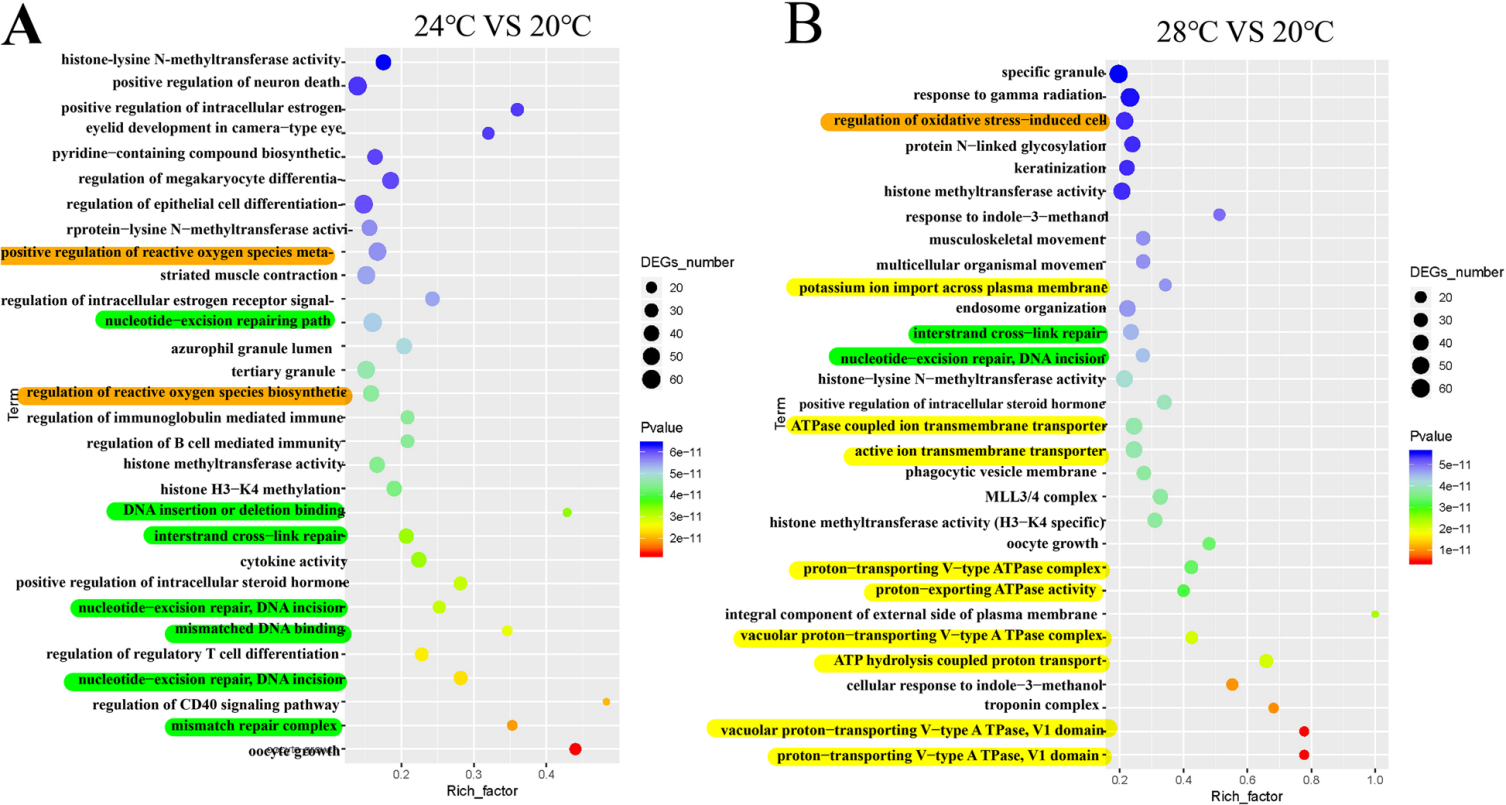
GO enrichment analysis of DEGs. GO enrichment analysis of DEGs identified in 24℃ vs 20℃ (**A**) and 28℃ vs 20℃ (**B**) groups, respectively. Osmoregulation-related terms are marked with yellow color; DNA-repair-related terms are marked with green color; apoptosis-related terms are marked with orange color

In 24℃ vs 20℃ group, top 30 GO terms were shown in Fig. 4A, mainly associating with DNA-repair and apoptosis, e.g., “mismatch repair complex”, “nucleotide-excision repair, DNA incision, mismatched DNA binding” and “regulation of reactive oxygen species biosynthetic process”. In 28℃ vs 20℃ group, top 30 GO terms relate to osmoregulation, DNA-repair and apoptosis, including “active ion transmembrane transporter activity”, “ATPase coupled active ion transmembrane transporter activity”, “nucleotide-excision repair, DNA incision,0 mismatched DNA binding” and “regulation of reactive oxygen species biosynthetic process” (Fig. 4B).

**Fig. 5:**
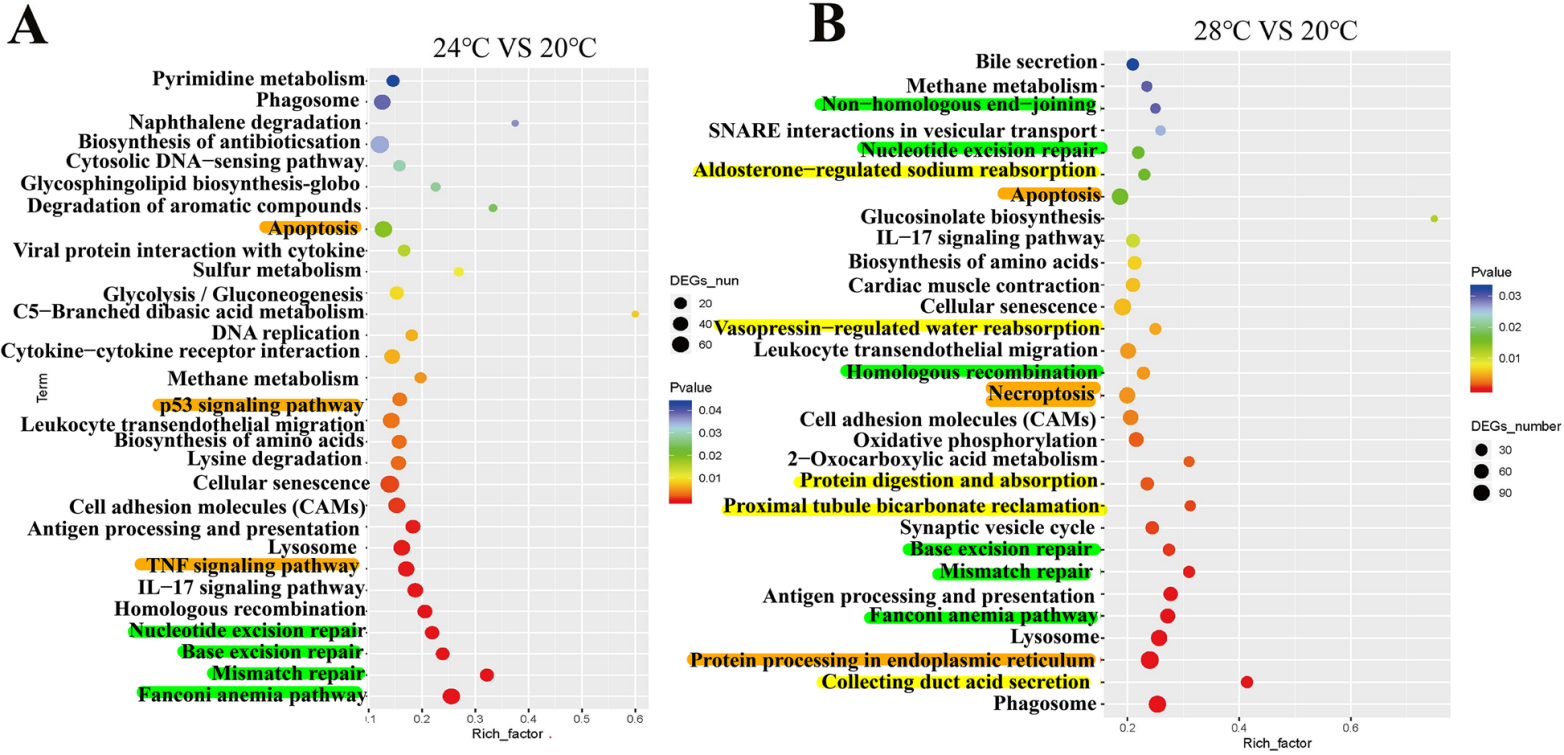
KEGG enrichment analysis of DEGs. KEGG enrichment analysis of DEGs identified in 24℃ vs 20℃ (**A**) and 28℃ vs 20℃ (**B**) groups, respectively. Osmoregulation-related terms are marked with yellow color; DNA-repair-related terms are marked with green color; apoptosis-related terms are marked with orange color

In KEGG enrichment analysis, top 30 KEGG pathways were presented in 24℃ vs 20℃ group, which involved in pathways of DNA repair (e.g., “Fanconi anemia pathway” “Mismatch repair” “Base excision repair” and “Nucleotide excision repair”) and apoptosis (e.g., “TNF signaling pathway” “p53 signaling pathway” and “apoptosis”) (Fig. 5A). In 28℃ vs 20℃ group, top 30 KEGG pathways included DNA repair related pathways, e.g., “Fanconi anemia pathway”, “Mismatch repair”, “Base excision repair”, “Homologous recombination”, “Nucleotide excision repair” and “Non − homologous end-joining”, apoptosis-related pathways, e.g., “Protein processing in endoplasmic reticulum”, “Necroptosis” and “Apoptosis” and osmoregulation-related pathways, e.g., “Collecting duct acid secretion”, “Proximal tubule bicarbonate reclamation”, “Protein digestion and absorption”, “Vasopressin-regulated water reabsorption” and “Aldosterone-regulated sodium reabsorption” (Fig. 5B).

### GO and KEGG enrichment analysis of osmoregulatory DEGs

**Fig. 6:**
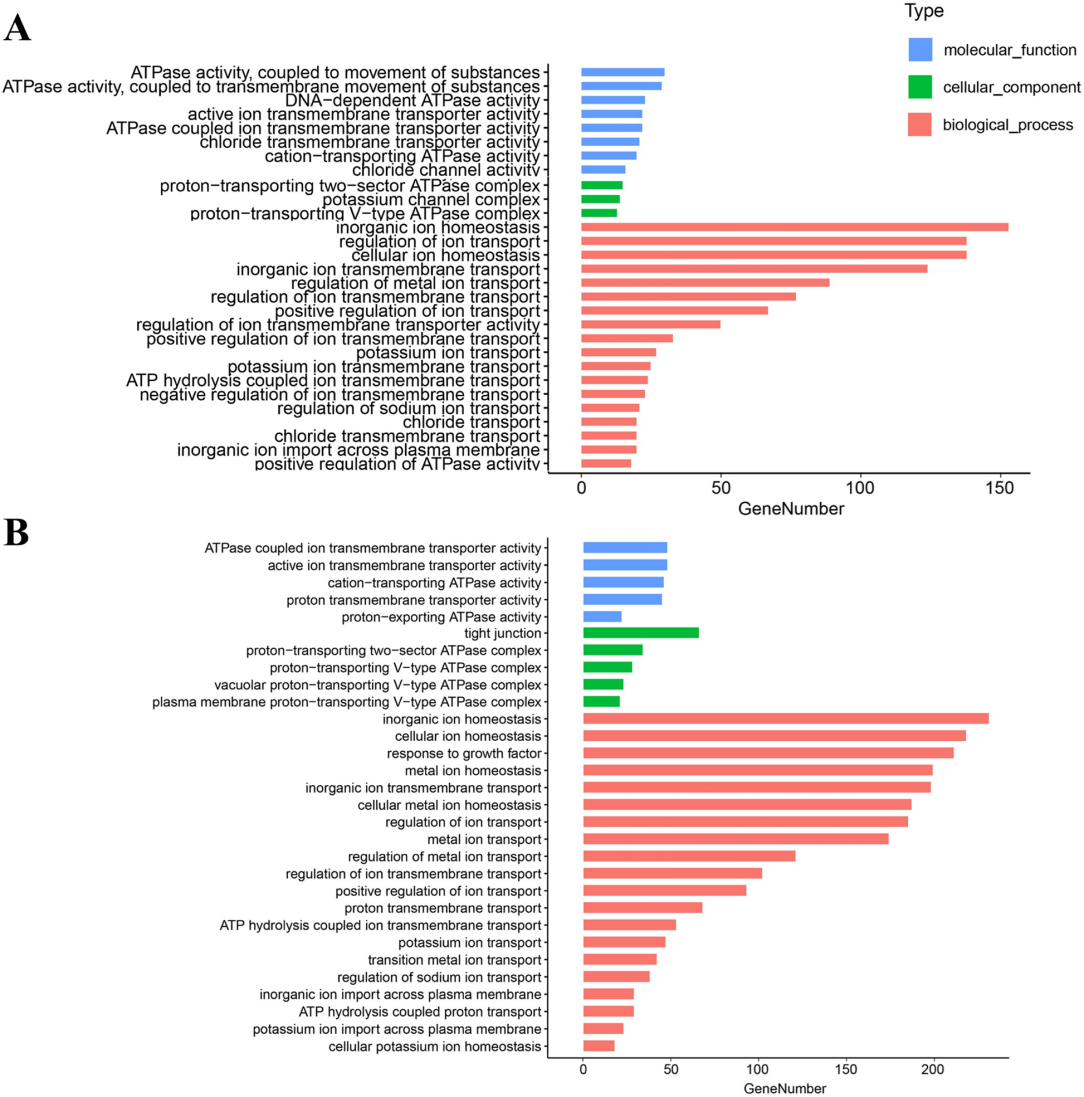
GO enrichment analysis of osmoregulatory DEGs in 24℃ vs 20℃ (**A**) and 28℃ vs 20℃ (**B**) groups. The DEGs were annotated into three gene ontology categories: biological process, molecular function, and cellular component

To investigate the effect of heat stress on osmoregulatory function, osmoregulatory DEGs were identified based on the genes enriched in the osmoregulatory terms of the GO enrichment analysis. In 24℃ vs 20℃ group, a total of 117 DEGs were composed of 92 up-regulated genes and 25 down-regulated genes. In 28℃ vs 20℃ group, 194 DEGs were identified, containing 172 up-regulated genes and 22 down-regulated genes. GO enrichment analysis showed that these DEGs were enriched into three major functional categories: biological processes (BP), cellular components (CC), and molecular functions (MF). In both 24℃ vs 20℃ and 28℃ vs 20℃ groups, many DEGs related to osmoregulation were enriched in “regulation of metal ion transport”, “inorganic ion homeostasis”, “cellular ion homeostasis”, “inorganic ion transmembrane transport”, and “regulation of ion transport” (Fig. 6).

In 24℃ vs 20℃ and 28℃ vs 20℃groups, top 10 KEGG pathways were shown in Fig. S3, of which mainly associate with osmoregulation function, such as “Tight junction”, “Collecting duct acid secretion”, “Vasopressin − regulated water reabsorption”, “Aldosterone − regulated sodium reabsorption” and “Endocrine and other factor-regulated calcium reabsorption”.

### Expression of osmoregulatory DEGs

**Fig. 7:**
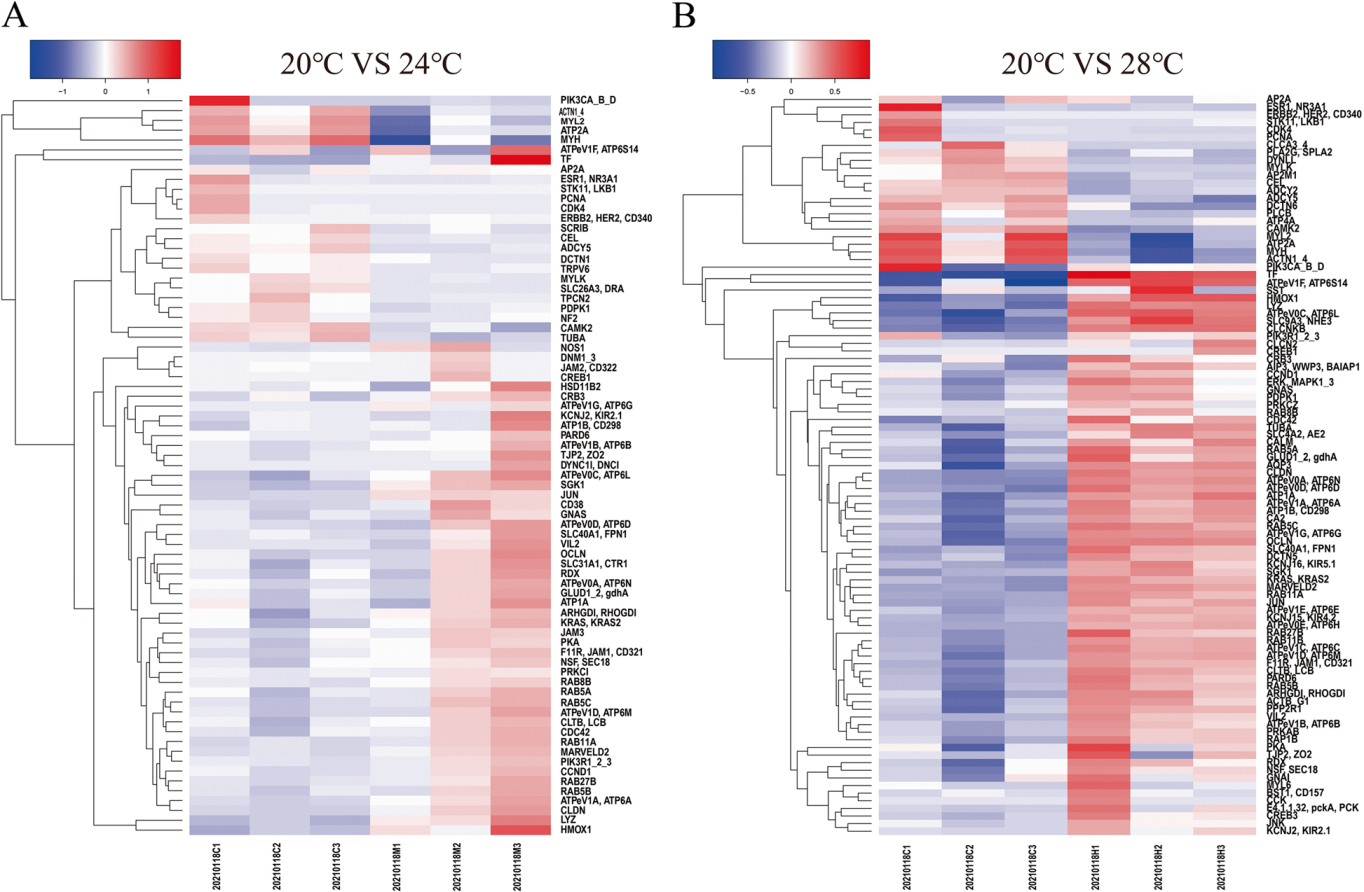
Heatmap of osmoregulatory DEGs in 24℃ vs 20℃ (**A**) and 28℃ vs 20℃ (**B**) groups

The expression of osmoregulatory DEGs at 24℃ vs 20℃ and 28℃ vs 20℃ groups were visualized by heat map (Fig. 7). Amongst, expression of seven ion absorption (*AE2, CLC-Kb, ATP1a, ATP2a, CLC-2, NHE3* and *ATP4a*), three resistance ion loss (*OCLN*, *JAM* and *CLDN*) and one water molecules' transport (*AQP3*) genes were changed after heat stress at 24℃ or 28℃. The seven ion absorption genes showed different expression changes, of which the expression level of *ATP2a* was significantly downregulated but upregulated in other genes, after heat stress at 24℃ or 28℃. The expression levels of three resistance ion loss genes were significantly increased at both 24℃ and 28℃, but the expression level of water molecules' transport gene was only increased at 28℃.

**Fig. 8:**
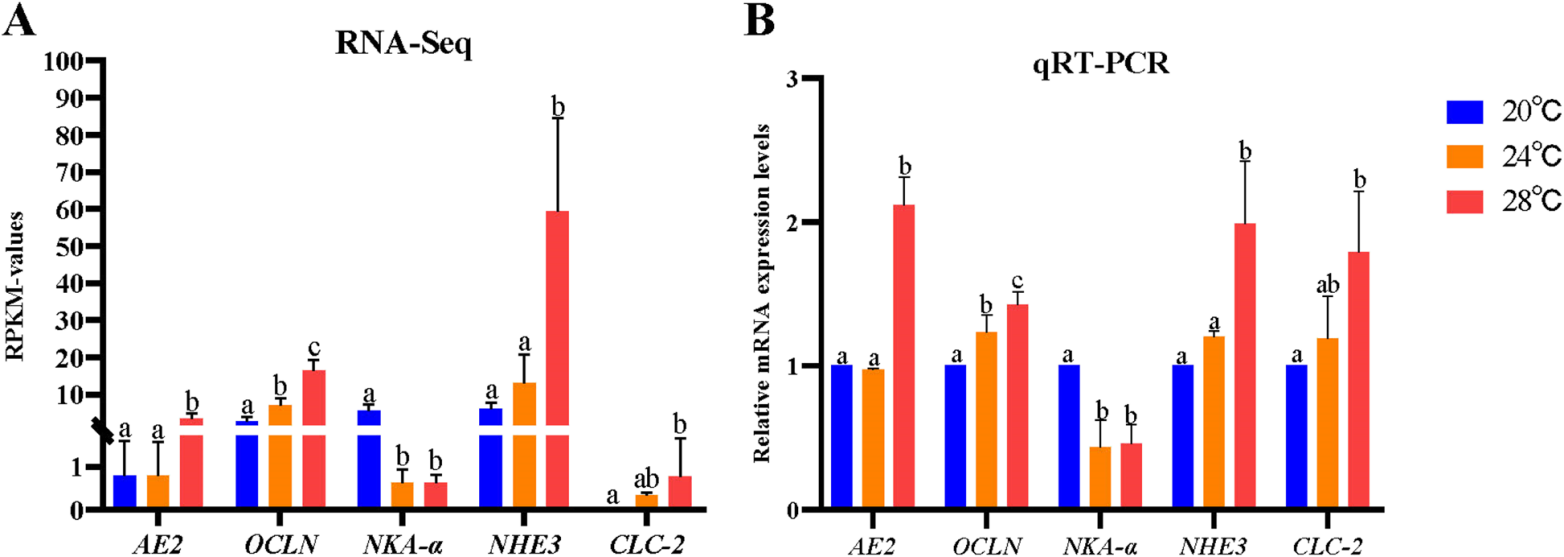
Validation of RNA-seq data. **A** and **B**: Comparison of the expression of 5 selected DEGs by RNA-seq and qRT-PCR. The gene expression of RNA-seq was presented based on the RPKM-values. The qPCR results were calculated by normalizing to the reference genes (*β-actin* and *EF-1α*), Mean ± SD. One-way ANOVA plus Bonferroni post-tests; different letters indicate statistically significant differences (*p* < 0.05)

The expression levels of 5 osmoregulatory DEGs were detected by qPCR in gill tissue after heat stress. As shown in Fig. 8B, the expression levels of *OCLN*, *NHE3*, *CLC-2* and A*E2* genes were upregulated, while the expression level of *NKA-a* gene was significantly downregulated at 24℃ or 28℃. The mRNA expression of these genes was consistent with the RNA-Seq results, indicating the transcriptome data is reliable.

### Discussion

Global warming has increased average summer temperatures and the frequency of extreme weather, posing the thermal threat to cold-water fishes [3]. Gill tissue directly contacts with the water environment and maintains the osmotic equilibrium in fishes [16, 18]. However, less information is available concerning the effects of heat stress on osmoregulation in gill tissue of cold-water fishes. To investigate the effect and mechanism of gill osmoregulation after heat stress in cold-water fishes, this study analyzed the changes of gill structure and ROS level, plasma ion concentration, and gill transcriptome in Siberian sturgeon.

The gill tissue is particularly sensitive to environmental temperature changes, making it be a perfect organ to study the effect of heat stress on cold-water fishes [20, 34]. In this study, we observed degeneration and hyperplasia of the gill tissue after heat stress at both 24℃ and 28℃, particularly at 28℃ where necrosis occurred. These results indicate that damage of gill tissue is emphasized with the increasing of heat stress temperature from 24℃ to 28℃, because the emergence of necrotic means possible dysfunction of the gill tissue [35]. Previous study has demonstrated that damage of gill tissue closely related to oxidative stress response [36]. Consistently, ROS level of gill tissue in Siberian sturgeon was upregulated after heat stress at both 24℃ and 28℃.

Na^+^, K^+^, and Cl^−^ are the major ions in the blood, and the change of their content has a great influence on the internal homeostasis of fish [10, 37]. The concentration of Na^+^ and K^+^ in the blood of Siberian sturgeon did not alter after the heat stress, while the content of Cl^−^ reduced dramatically at 28 °C compared to the control group (Fig. 3). However, no significant changes of Na^+^, K^+^, and Cl^−^ concentration were observed in blood ions of European seabass (*Dicentrarchus labrax*) and Atlantic salmon (*Salmo salar*) exposed to rising temperature [4, 26, 38], which is different from our results. We speculate that it may be related to kidney damage in fish. Kidney is the organ of Cl^−^ reabsorption in fish [37]. Moreover, renal tissue damage can affect the expression of chloride channel genes [39].

Temperature is an important environmental factor affecting the physiological function of fish [40–42]. Osmotic regulation is one of the important physiological functions to maintain fish survival [12, 37]. The GO analysis of DEGs showed that heat stress at 24℃ and 28℃ activated gene expression enriched into “regulating ion transport” (Fig. 6). KEGG pathway enrichment analysis showed that “Tight junction” was enriched (Fig. S3). Tight junctions are essential for establishing a selectively permeable barrier to diffuse ions through the paracellular space between neighboring cells [43]. Previous studies demonstrated that activating Tight junction pathway could reduce ion loss in gill tissue [17, 44]. Thus, our results indicate that heat stress may affect the osmotic function of gill tissue.

To maintain ion equilibrium in fresh water, gill tissue performs osmotic regulation by lowering passive ion outflow and actively absorbing ions from water [12, 14]. Five genes related to fish osmosis were selected to qPCR analysis from the DEGs. OCLN, one of integral membrane proteins, constitutes the chordates zonula occludes, and it plays an important role in osmotic regulation of fish [43]. In our results, *OCLN* expression was increased at 24℃ and 28℃ (Fig. 8B). When fish migrate from seawater to freshwater, gill tissue resists ion loss by increasing *OCLN* expression [17, 45, 46]. At the same time, the expression levels of the ion absorption genes (*NHE3*, *CLC2*, *AE2*) tended to increase with temperature rising (Fig. 8B). It has been demonstrated that the gill tissue improves active ion absorption capacity and resists ion loss by increasing the expression levels of *NHE3*, *CLC-2*, and *AE2,* when fishes migrate from high-salt environment to freshwater [15, 47–49]. Numerous previous studies have shown that *NKA-α* expression decreases when water temperature increases [22, 50, 51], this was substantiated by the change results of heat stress at 28℃ and 24℃ when compared to 20℃ in our study (Fig. 8B).

**Fig. 9:**
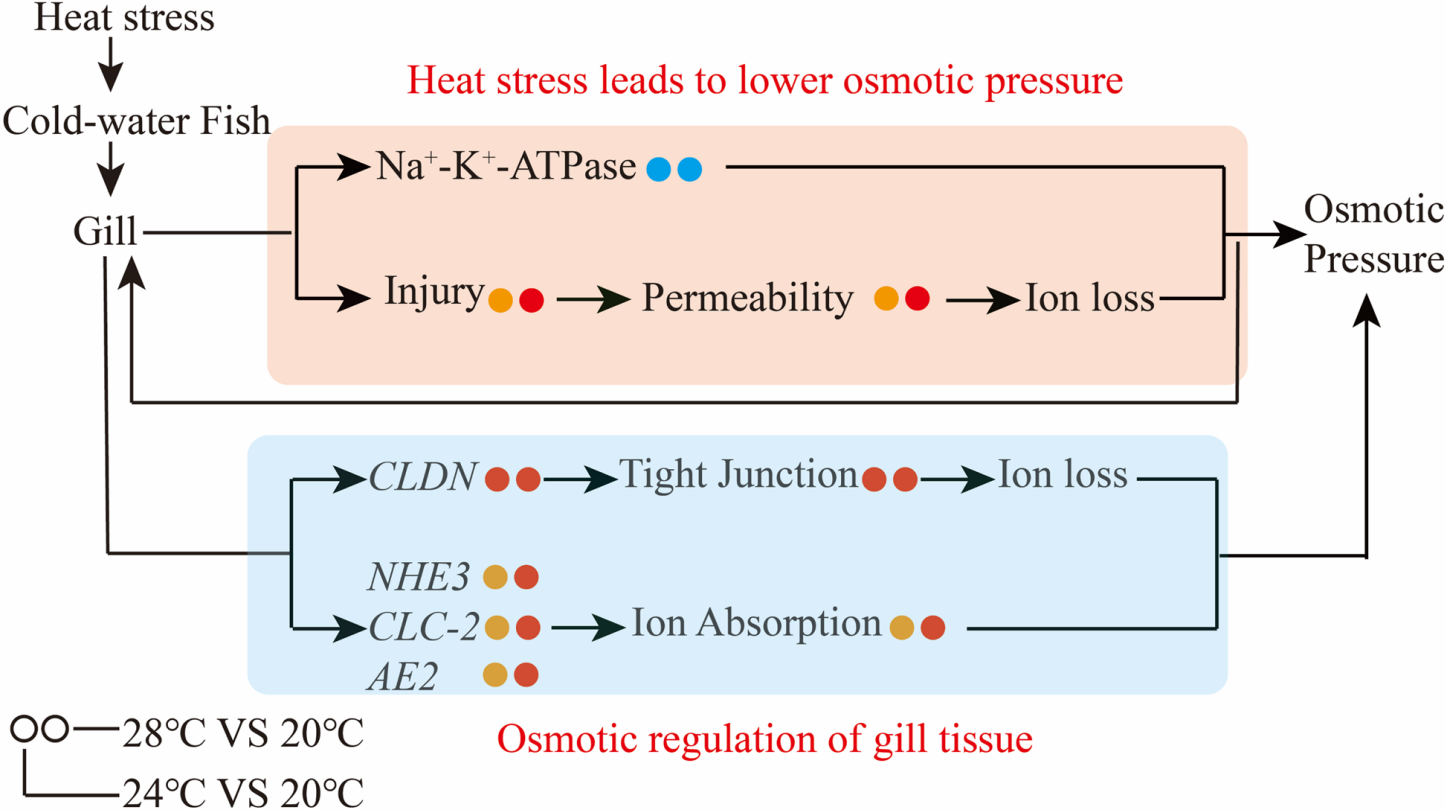
Graphic represents the effects of heat stress on the osmotic function of gill tissue in cold-water fishes. Orange box indicates heat stress-induced lower osmotic pressure in gill tissue, blue box means the osmotic regulation of gill tissue against the heat stress. The dots on left and right sides present the gene expression change in 24℃ vs 20℃ and 24℃ vs 20℃ groups, respectively. Red, blue and orange dots suggest significant upregulation (*p* < 0.05), downregulation (*p* < 0.05), and change without significant difference (*p* > 0.05), respectively

In summary, our study reveals that heat stress can regulate osmotic function of gill tissue in cold-water fishes. As shown in Fig. 9, we speculate that heat stress results in gill damage and decreases activity of Na^+^-K^+^-ATPase, which may further increase the permeability of fish’s gill tissue and boost fish ion loss in fresh water. Besides, to reduce ion loss, the gill tissue keeps ion balance through active ion absorption and passive ion outflow. When gill tissue is slightly damaged, it can withstand ion loss by upregulating *CLDN* gene expression and increasing the tightness of the tissue's connections. Otherwise, when gill tissue is severely damaged and permeability increases, gill tissue will activate *NHE3*, *AE2*, *CLC-2* to absorb ions from water and, thereby, maintain the in vivo osmotic equilibrium.

## Materials and Methods

### Fish breeding

A total of 300 healthy Siberian sturgeon (length of 25.0 ± 2.3 cm, weight of 86.0 ± 4.0 g) were purchased from Sichuan Runzhao Fisheries Co., Ltd., Sichuan, China. All fishes were bred in 40 × 80 × 50 cm^3^ indoor tanks with an average temperature of 20℃. The sturgeons were fed with 1% body weight of commercial feed (Haida Group Co., Ltd, Guangdong, China) three times per day (at 8:00 am, 14:00 pm, and 20:00 pm, respectively) and bred at least one week of acclimatization before temperature administration. The handling of animals was performed in accordance with the protocols approved by the Animal Protection Committee of Sichuan Agricultural University.

### Heat stress administration

After one week of acclimatization, 270 fishes were randomly divided into three groups (*n* = 90): the control group (at 20℃) and two heat stress groups (at 24℃ and 28℃, respectively). The fishes of two heat stress groups underwent an increasing temperature of 1 ± 0.5℃/day within 8 days to 24℃ and 28℃, respectively, then were maintained at the heat stress temperature for 12 days (Fig. S1).

### Sampling

Thirty fishes were randomly selected from each group and anesthetized by 200 mg/L MS-222 (yuxi biological technology co., Lianyungang, China). Blood was collected in tube with heparin sodium for detection of plasma ion contents, and gill tissues were sampled for histopathological observation, ROS immunofluorescence, transcriptome analysis, and qPCR analysis.

### Histopathological observation

The acquired gill tissues were fixed with 10% formaldehyde solution for at least 24 h, then dehydrated in graded ethanol solutions, cleared in xylene, embedded in paraffin wax and sectioned into 3–5 μm sections. Sections were prepared, then mounted on slides for hematoxylin and eosin (H&E). After staining, the sections were observed and imaged by a Motic BA400Digital microscope (Motic China Group, Co., Ltd, Xiamen, China).

### ROS immunofluorescence

The refrigerant gill tissues were embedded by OCT (Sakura, USA), then sliced by freezing microtome (Thermo, CRYOSTAR NX50, US). The slice thickness was 8–10 μm. The frozen slides were restored to room temperature, then incubated with spontaneous fluorescence quenching reagent (Servicebio, China). ROS staining solution (Servicebio, Wuhan, China) was used to stain slides at 37℃ for 30 min in dark place, following washing three times with PBS (pH 7.4) and then incubating with DAPI solution (Servicebio, Wuhan, China) at room temperature for 10 min in dark place. Finally, the sections were observed by Fluorescent Microscopy (Pannoramic MIDI, 3DHISTECH, HUN).

### Determination of plasma Na^+^, K^+^ and Cl^−^ contents

The blood was centrifuged at 4000 × g for 10 min, the plasma was collected and frozen at -80℃ until for use. Plasma Na^+^, Cl^−^ and K^+^ contents were measured by detection kits (Nanjing Jiancheng Bioengineering Institute, Nanjing, China). All the procedures were performed following strict adherence to the manufacturer’s instructions.

### RNA extraction, library construction, and Illumina sequencing

Total RNA was extracted from the gill tissue by TRIzol® Reagent according to the manufacturer’s instructions (Invitrogen, Carlsbad, CA, USA), and genomic DNA was removed by DNase I (Takara, Dalian, China). Then, RNA quality was determined by 2100 Bioanalyser (Agilent Technologies, Santa Clara, CA, USA)) and quantified by ND-2000 (NanoDrop Technologies, Shanghai, China). The high-quality RNA sample (OD260/280 = 1.8 ~ 2.2, OD260/230 ≥ 2.0) was used to construct sequencing libraries. RNA-seq transcriptome libraries were prepared following TruSeqTM RNA sample preparation Kit from Illumina (San Diego, CA, USA). Libraries were selected for cDNA target fragments of 200–300 bp on 2% Low Range Ultra Agarose followed by PCR amplified using Phusion DNA polymerase (NEB) for 15 PCR cycles. After quantified by TBS380, paired-end libraries were sequenced by Illumina NovaSeq 6000 platform (Shanghai BIOZERON Co., Ltd).

### *De novo* assembly and annotation

The raw paired-end reads were firstly trimmed, and quality was controlled by Trim Galore and Fast QC software. Then, clean data from all samples was used to *de novo* assembly with Trinity (http://trinityrnaseq.sourceforge.net/). All the assembled transcripts were annotated against NR, GO, eggNOG, KEGG and Swiss databases. NR database was used to identify the proteins that have the highest sequence similarity with the given transcripts. BLAST2GO (http://www.blast2go.com/b2ghome) program was used to get GO annotations of unique assembled transcripts for describing biological processes, molecular functions, and cellular components.

### DEGs identification, GO and KEGG enrichment analysis

After assembly, the gene expression abundance was calculated as FPKM (Fragments Per Kilobase of transcript per Million mapped reads) values that were used to analyze the expression change of genes in the gill tissues of Siberian sturgeon after heat stress. The DEGs of heat stress groups than the control group were identified via identified by DEseq R packages within adjusted *p* < 0.005, |log_2_ (foldchange)|≥ 2. The acquired DEGs then underwent GO and KEGG enrichment analysis [52–54].

### Verification by quantitative real-time PCR (qRT-PCR)

5 DEGs were selected to verify the RNA-Seq results. These genes were measured by qRT-PCR. The Primer 6.0 software was used to design primers (Table 2). Total RNA was isolated from the gill with an animal tissue total RNA extraction kit (Fuji, Chengdu, China). cDNA was synthesized from 2 μg of RNA using a RT Easy™ II kit (Fuji). qPCR was performed using a SYBR green real-time PCR kit (Takara, Kyoto, Japan) and a Thermo Cycler (BioRad, Hercules, CA, USA).

**Table 2:** Primer used for RT-qPCR

Sequence name	Annotation	Sequence (5′-3′)	Product size (bp)	Tm (℃)
Reference gene	*β -actin*	F: TGGACGCCCAAGACATCAGG	127	59.6
		R: GGTGACAATGCCGTGCTCG		
Reference gene	*EF-1α*	F: TGAAGGCTGGCATGATCGTC	82	58.8
		R: AGGGTCTCGTGGTGCATTTC		
TRINITY_DN146301_c2_g3	*AE2*	F: CGTTCGTGCGTCTGAAGGAT	127	58.6
		R: GTCTGCCATGAGAGTGGAGATG		
TRINITY_DN133105_c0_g5	*OCLN*	F: GCTCCGCCTTCTACAACCAAG	173	56.8
		R: GCCAATGCCACAGTGACGAT		
TRINITY_DN111957_c0_g1	*NKA-α*	F: CTCCAAGCCAGAGGTTAGCG	109	57.6
		R: CACTTGAGACGAGGAACATTGC		
TRINITY_DN142433_c1_g1	*NHE3*	F: GACAACCACACCAACGACTCT	275	58.6
		R: GTCCTACCTAACGGCAGAGATG		
TRINITY_DN141795_c0_g2	*CLC-2*	F: GCTTGTGGATCTCCGTGGTCTC	97	59.2
		R: AGCTGAGGAAGGCGATTGAAGG		

To distinguish between specific and nonspecific reaction products, a melting curve was obtained at the end of each run (Table 2). The 2^−△△Ct^ method was used to calculate relative changes in mRNA transcript expression from the qPCR results (ΔCT = CT target gene – Ct reference gene, ΔΔCT = ΔCT experimental—ΔCT control).

### Data analysis

All data were expressed as mean ± standard deviation. Differences between groups were analyzed by one-way analysis of variance (ANOVA) followed by Bonferroni’s Multiple Comparison Test with the significance as *p* < 0.05.

## Supplementary Information

**Additional file 1: Fig.S1.** Temperature control Day1 to 7: temperature acclimation; Day7 to 14: Reaching targeted temperature. Day15 to 27: Summer water temperature exposure. **Fig. S2.** DEGs analysis. (A, B) DEGs analyzed in 24℃-vs-20℃. (C, D) DEGs in 28℃-vs-20℃. Each dot represents one gene. Red dots represent up-regulated genes and blue dots represent down-regulated genes. Gray dots represent genes with no differential expression. **Fig. S3.** KEGG pathway of osmoregulation DEGs. (A) 24℃-vs-20℃. (B) 28℃-vs-20℃. **Table S1.** The sequence quality and mapping results in the nine samples.

## Data Availability

The datasets generated and analyzed during the current study are available in the Sequence Read Archive of National Center for Biotechnology Information database with accession number PRJNA765171 (https://dataview.ncbi.nlm.nih.gov/object/PRJNA765171). The datasets analyzed during this study are included in this published article and its supplementary information files. Please contact Shiyong Yang (yangshiyong@sicau.edu.cn) if someone wants to request the data from this study.

## Abbreviations

qRT-PCR: Quantitative real-time PCR
GO: Gene Ontology
BP: Biological process
CC: Cellular component
KEGG: Kyoto Encyclopedia of Genes and Genomes
