# Supplementary material for: Transcriptome analysis reveals the high temperature induced damage is a significant factor affecting the osmotic function of gill tissue in Siberian sturgeon (Acipenser baerii)
Source: BMC Genomics. 2023 Jan 3;24:2. doi: 10.1186/s12864-022-08969-9 (PMC9809011; doi:10.1186/s12864-022-08969-9)
Supplement: Supplementary file 1 — Additional file 1: Fig.S1. Temperature control Day1 to 7: temperature acclimation; Day7 to 14: Reaching targeted temperature. Day15 to 27: Summer water temperature exposure. Fig. S2. DEGs analysis. (A, B) DEGs analyzed in 24℃-vs-20℃. (C, D) DEGs in 28℃-vs-20℃. Each dot represents one gene. Red dots represent up-regulated genes and blue dots represent down-regulated genes. Gray dots represent genes with no differential expression. Fig. S3. KEGG pathway of osmoregulation DEGs. (A) 24℃-vs-20℃. (B) 28℃-vs-20℃. Table S1. The sequence quality and mapping results in the nine samples. [file 12864_2022_8969_MOESM1_ESM.zip › Supplementary Material/Table S1.docx]

**Table S1**

The sequence quality and mapping results in the nine samples.

| Library | Clean Reads | Mapped Reads | Mapping Rate (%) | %≥Q30 |
| --- | --- | --- | --- | --- |
| 20℃ 1 | 44375520 | 31899830 | 71.88 | 94.29 |
| 20℃ 2 | 30704356 | 21898469 | 71.32 | 95.29 |
| 20℃ 3 | 32013344 | 22923186 | 71.60 | 95.73 |
| 24℃ 1 | 41113914 | 30738982 | 74.76 | 95.74 |
| 24℃ 2 | 34034488 | 23514938 | 69.09 | 95.63 |
| 24℃ 3 | 39977378 | 28592620 | 71.52 | 95.88 |
| 28℃ 1 | 43665210 | 31369785 | 71.84 | 95.96 |
| 28℃ 2 | 30650902 | 21560855 | 70.34 | 95.94 |
| 28℃ 3 | 32157052 | 22578527 | 70.21 | 96.04 |
