# Supplementary figures and images for: Transcriptome analysis reveals the high temperature induced damage is a significant factor affecting the osmotic function of gill tissue in Siberian sturgeon (Acipenser baerii)
Source: BMC Genomics. 2023 Jan 3;24:2. doi: 10.1186/s12864-022-08969-9 (PMC9809011; doi:10.1186/s12864-022-08969-9)

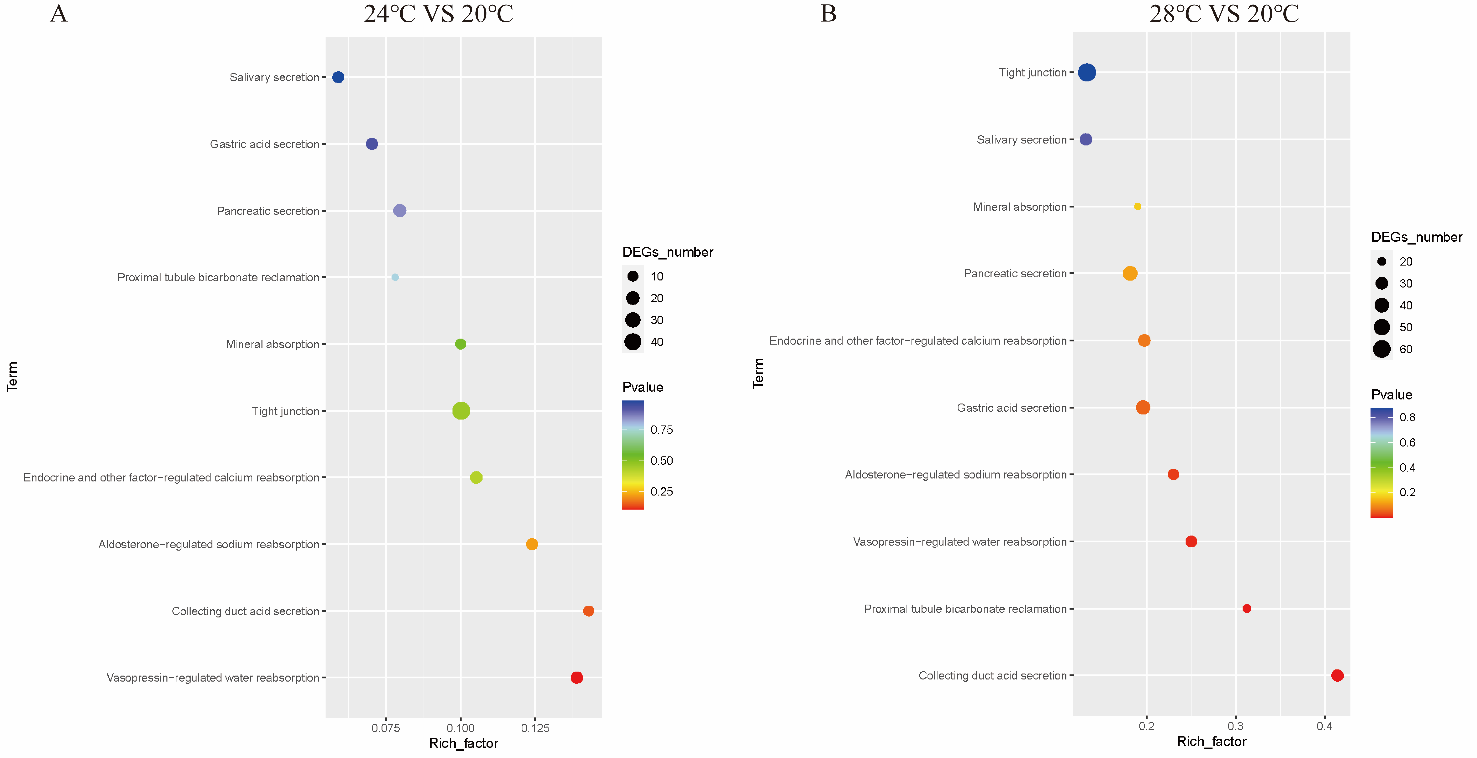


**Fig. S3.** KEGG pathway of osmoregulation DEGs. (A) 24℃-vs-20℃. (B) 28℃-vs-20℃.

Supplement: Supplementary file 1 — Additional file 1: Fig.S1. Temperature control Day1 to 7: temperature acclimation; Day7 to 14: Reaching targeted temperature. Day15 to 27: Summer water temperature exposure. Fig. S2. DEGs analysis. (A, B) DEGs analyzed in 24℃-vs-20℃. (C, D) DEGs in 28℃-vs-20℃. Each dot represents one gene. Red dots represent up-regulated genes and blue dots represent down-regulated genes. Gray dots represent genes with no differential expression. Fig. S3. KEGG pathway of osmoregulation DEGs. (A) 24℃-vs-20℃. (B) 28℃-vs-20℃. Table S1. The sequence quality and mapping results in the nine samples. [file 12864_2022_8969_MOESM1_ESM.zip › Supplementary Material/Figure S3.docx]
